# Mediating oxidative stress enhances α-ionone biosynthesis and strain robustness during process scaling up

**DOI:** 10.1186/s12934-022-01968-1

**Published:** 2022-11-23

**Authors:** Ching-Ning Huang, Xiaohui Lim, Leonard Ong, Chinchin Lim, Xixian Chen, Congqiang Zhang

**Affiliations:** Singapore Institute of Food and Biotechnology Innovation (SIFBI), Agency for Science, Technology and Research (A*STAR), 31 Biopolis Way, Level 6, Nanos Building, Singapore, 138669 Singapore

**Keywords:** Ionone, Apocarotenoids, Carotenoid cleavage dioxygenases (CCDs), Reactive oxygen species (ROSs), Hydrogen peroxide (H_2_O_2_), Alkyl hydroperoxide reductases

## Abstract

**Background:**

α-Ionone is highly valued in cosmetics and perfumery with a global usage of 100–1000 tons per year. Metabolic engineering by microbial fermentation offers a promising way to produce natural (*R*)-α-ionone in a cost-effective manner. Apart from optimizing the metabolic pathways, the approach is also highly dependent on generating a robust strain which retains productivity during the scale-up process. To our knowledge, no study has investigated strain robustness while increasing α-ionone yield.

**Results:**

Built on our previous work, here, we further increased α-ionone yield to 11.4 mg/L/OD in 1 mL tubes by overexpressing the bottleneck dioxygenase CCD1 and re-engineering the pathway, which is  > 65% enhancement as compared to our previously best strain. However, the yield decreased greatly to 2.4 mg/L/OD when tested in 10 mL flasks. Further investigation uncovered an unexpected inhibition that excessive overexpression of CCD1 was accompanied with increased hydrogen peroxide (H_2_O_2_) production. Excessive H_2_O_2_ broke down lycopene, the precursor to α-ionone, leading to the decrease in α-ionone production in flasks. This proved that expressing too much CCD1 can lead to reduced production of α-ionone, despite CCD1 being the rate-limiting enzyme. Overexpressing the alkyl hydroperoxide reductase (*ahpC/F*) partially solved this issue and improved α-ionone yield to 5.0 mg/L/OD in flasks by reducing oxidative stress from H_2_O_2_. The strain exhibited improved robustness and produced  ~ 700 mg/L in 5L bioreactors, the highest titer reported in the literature.

**Conclusion:**

Our study provides an insight on the importance of mediating the oxidative stress to improve strain robustness and microbial production of α-ionone during scaling up. This new strategy may be inspiring to the biosynthesis of other high-value apocarotenoids such as retinol and crocin, in which oxygenases are also involved.

**Supplementary Information:**

The online version contains supplementary material available at 10.1186/s12934-022-01968-1.

## Background

Ionones are a group of natural ketone compounds composed of 13 carbons with a monocyclic terpenoid backbone [1]. They are carotenoid-derived aroma compounds found at sub-ppm levels in many flowers and fruits (e.g., rose, sweet osmanthus, orris root, and raspberry). Among these, α-ionone is a high-value aroma (violet-like) and flavor (raspberry and blackberry-like) [2] compound with an extremely low odor threshold (0.4–3.2 ppb) [3] and a high global usage (on a scale of 100–1000 tons per year). Commercial α-ionone is currently chemically synthesized, as the extremely low levels in natural sources (e.g., 100 tons of raspberries yields merely 1 g of α-ionone) makes such extraction commercially unviable [4].

**Fig. 1:**
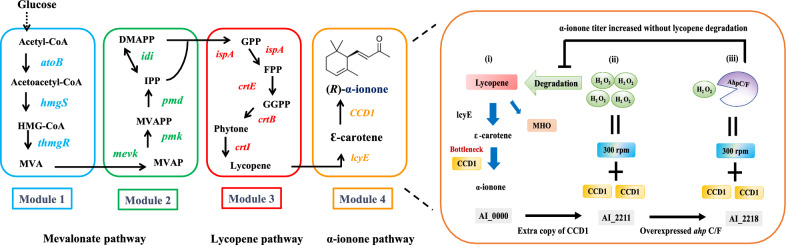
The metabolic pathway and the proposed scheme illustrating how CCD1 overexpression affects α-ionone production by oxidative stress. (i) The pathway with low oxidative stress, namely strain AI_0000. (ii) The pathway with high oxidative stress, namely strain AI_2211. (iii) The pathway with low oxidative stress due to H_2_O_2_ elimination by AhpC/F, namely strain AI_2218

α-Ionone is the oxidative product of α-carotene or ε-carotene that is catalyzed by carotenoid cleavage dioxygenases (CCDs) [5]. CCDs can cleave multiple substrates and typically exhibit a high degree of regiospecificity for double bond positions. In a previous study, we designed an *E. coli*-based “plug-n-play” system to produce α-ionone, β-ionone and retinoids. We fused the carotenoid cleavage dioxygenase 1 (CCD1) from *Osmanthus fragans* (OfCCD1) with thioredoxin (trxA) to form TOfCCD1 for increasing CCD1 enzyme concentration in the pathway to achieve high titers (this strain was named AI_0000 (Fig. 1)) [4]. Nevertheless, ε-carotene (the substrate of CCD1) was still not completely converted in the bacterial strain. This limitation might be because of the poor catalytic properties of TOfCCD1 [4]. To address this issue, we applied directed evolution and enzyme fusion to further engineer the TOfCCD1 enzyme. We successfully improved CCD1 activity and further enhanced α-ionone production, achieving approximately 3.5 mg/L/OD of α-ionone. [6].

In this study, we further improved the titer of α-ionone by overexpressing an additional copy of CCD1 on the vector to enhance ε-carotene conversion to α-ionone (strain AI_2211, Fig. 1). However, we found that oxidative stress hindered the ionone production especially for our newly engineered strains when mixing speed was increased during scaling up. This instability severely impacts further scaling up to bioreactors. As oxidative stress is related with reactive oxygen species (ROSs) in *E. coli*, we hypothesized that this phenomenon may be related to the generation of ROSs, particularly hydrogen peroxide (H_2_O_2_). By overexpressing a catalase G (*katG*) or an alkyl hydroperoxide reductase (*ahpC/F*), we found that AhpC/F could effectively remove H_2_O_2_ in our bacterial strain and hence increased the ionone titer (strain AI_2218, Fig. 1). The strain overexpressing AhpC/F was successfully scaled up in 5L bioreactors and achieved approximately 700 mg/L α-ionone, the highest titer in literature.

## Results

### Oxygen sensitivity of new strain: AI_2211

**Fig. 2:**
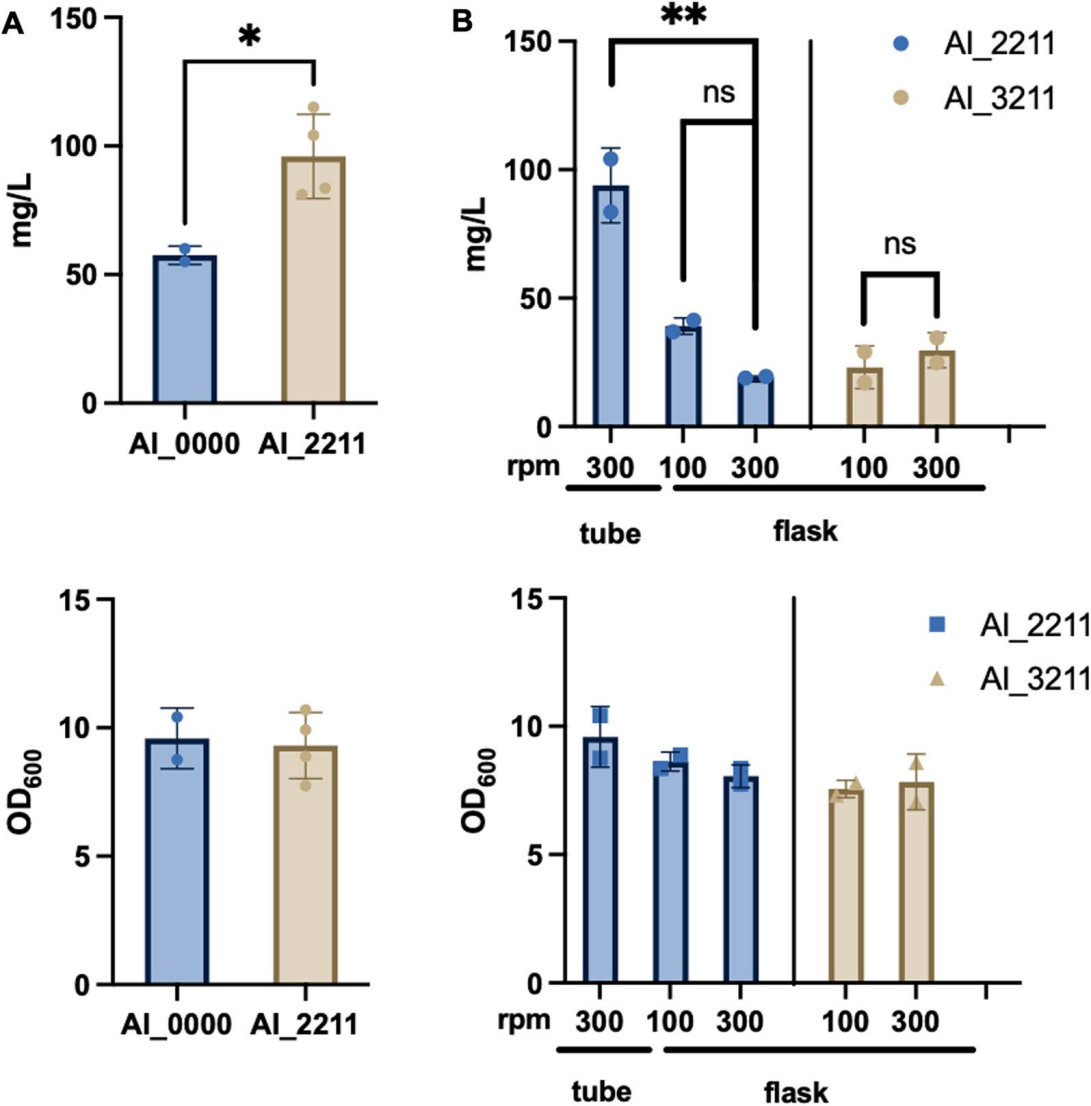
Titer (mg/L) and Optical density (OD_600_) of α-ionone production by different strains. **A** Strains AI_0000 and AI_2211 were cultured in the 1 ml defined medium, shaken at 300 rpm in snap-cap tube. **B** Strain AI_2211 were cultured under the conditions with 1 ml defined medium, shaken at 300 rpm in snap-cap tubes or 10 ml defined medium, shaken at 100 or 300 rpm in flasks. Strain AI_3211 were cultivated in the 10 ml defined medium in shaking flasks at 100 or 300 rpm. The data were an average of duplicate data. The titers were statistically analyzed by one-way ANOVA: ns, P > 0.05; * P < 0.05; ** P < 0.01; *** P < 0.001

Previously, we constructed a modular biosynthetic pathway for α-ionone production in *E. coli* [4]. This biosynthetic pathway consists of 4 modules: (I) an upstream mevalonate pathway; (II) a downstream mevalonate pathway; (III) a lycopene pathway; and (IV) an α-ionone pathway (Fig. 1). Based on our modification of this biosynthetic pathway, we named the α-ionone producing strain AI_ABCD (Additional file 1). To improve the production of α-ionone, we overexpressed an additional copy of CCD1 in the first module to enhance the ε-carotene conversion to α-ionone and the expression levels of pathway genes were re-optimized, namely, strain AI_2211 (Fig. 1) [7]. We first tested the strain in snap-cap tubes (with the shaking speed of 300 rpm) and the strain AI_2211 produced 96 mg/L of α-ionone, which is  > 65% higher than our previous best strain AI_0000 (Fig. 2A). However, the titer dropped to 19.3 mg/L when we tested the strain in flasks (with the shaking speed of 300 rpm) (Fig. 2B). In flasks, α-ionone production increased from 19.3 to 39.2 mg/L while reducing the shaking speed from 300 to 100 rpm (Fig. 2B). This indicated that the productivity of strain AI_2211 was highly sensitive to the change of shaking speed. We wondered if the observed negative effect of shaking speeds on α-ionone production was due to an additional copy of the CCD1 gene on the plasmids present in strain AI_2211. To test this hypothesis, we removed the extra CCD1 gene while keeping all the remaining genes identical to strain AI_2211 in obtaining strain AI_3211 (Additional file 1). We compared these two strains at shaking speeds of 100 rpm and 300 rpm in flasks. Interestingly, strain AI_3211 produced a slightly higher amount of α-ionone at 300 rpm (30 mg/L) compared to at 100 rpm (23 mg/L), suggesting the strain was less sensitive to the change of shaking speed (Fig. 2B). In contrast, strain AI_2211 produced around a twofold higher amount of α-ionone at 100 rpm compared to 300 rpm (Fig. 2B). Higher shaking speeds contributed to higher amounts of dissolved oxygen and potentially higher oxidative stress in flasks. Hence, we hypothesized that this phenomenon might be attributed to the generation of reactive ROSs, especially H_2_O_2_.

### H_2_O_2_ production of in vivo overexpression of catalase genes; catalase G (katG): strain AI_2217 and alkyl hydroperoxide reductase (ahpC/F): strain AI_2218

**Fig. 3:**
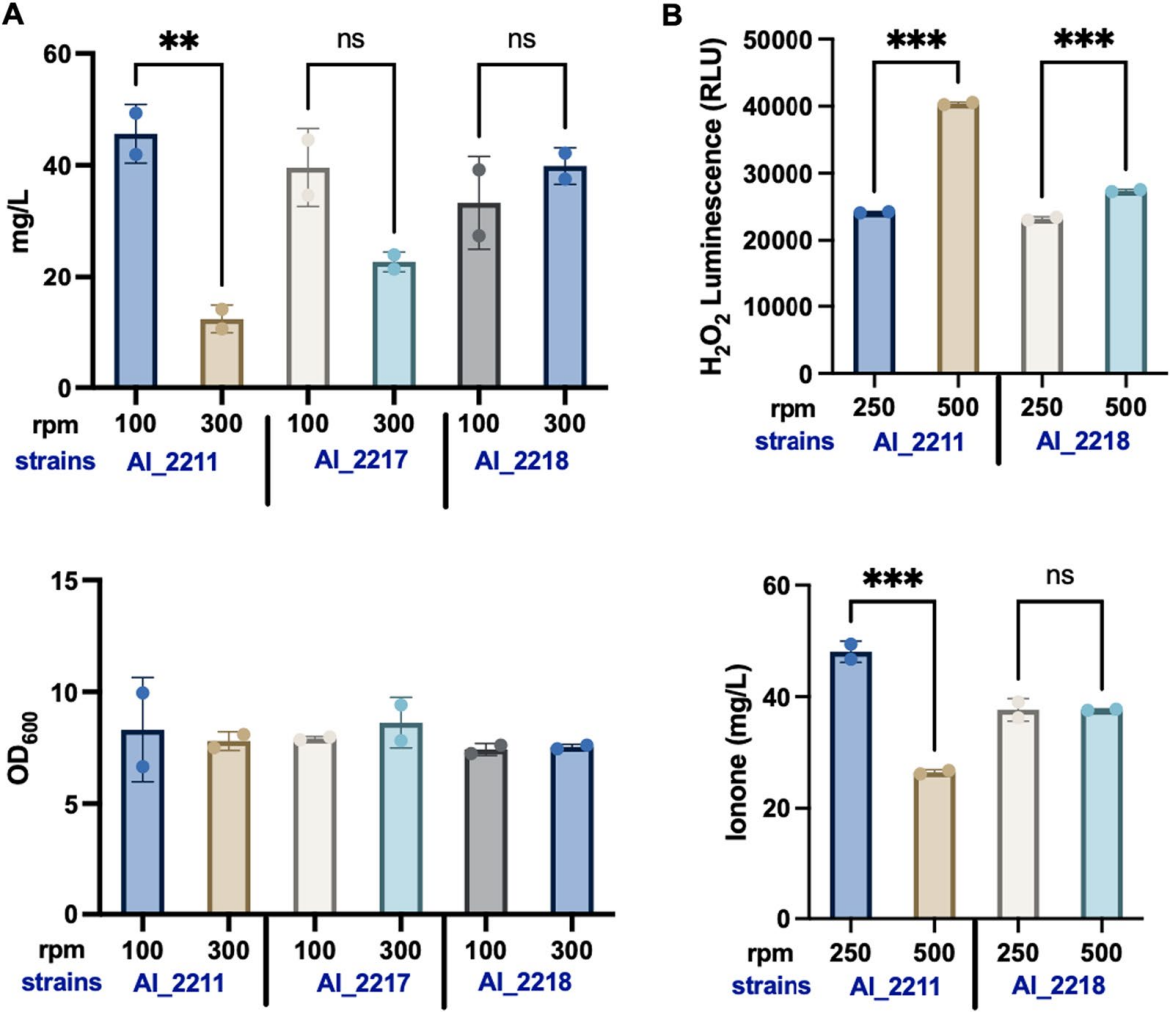
In vivo overexpression of *katG* and *ahpC/F* and ROS-GloTM Assay signals from H_2_O_2_ production by strains. **A** Strains AI_2211, AI_2217, and AI_2218 were cultivated in the 10 ml defined medium with 10 ml dodecane in shaking flasks at 100 rpm and 300 rpm. The data were an average of duplicate data. **B** α-Ionone titers of AI_2211 and AI_2218 under 250 rpm or 500 rpm shaking speed by RTS-1C (Personal bioreactor) and after incubation 1.5 h, 100 µL of each testing strains AI_2211 and AI_2218 mixed culture plated in a 96-well white cell culture plate and 100 µL of ROS-GloTM Detection Solution was added to the wells. Luminescence was determined with a GloMax^®^ Multi + Luminometer. The average relative light unit (RLU) and standard deviation of quadruplicate samples were calculated. The titers and luminescence values were statistically analyzed by one-way ANOVA: ns, P > 0.05; * P < 0.05; ** P < 0.01; *** P < 0.001

To test our hypothesis, we overexpressed *katG* and *ahpC/F* in strain AI_2211 to obtain strain AI_2217 and AI_2218, respectively (Additional file 1). We then compared α-ionone titers of 3 different strains (AI_2211, AI_2217, and AI_2218). We found strain AI_2218 yielded higher α-ionone titers at higher shaking speeds (300 rpm vs 100 rpm). However, strain AI_2217 exhibited the same trend as strain AI_2211 with about a twofold higher yield of α-ionone at 100 rpm compared to that at 300 rpm (Fig. 3A). The results showed that α-ionone titers of strains AI_3211 and AI_2218 were positively correlated with shaking speed and relatively insensitive to the change of shaking speed, while those of strains AI_2211 and AI_2217 were negatively correlated with shaking speed and displayed higher sensitivity towards shaking speed change (Figs. 2B and 3A). Furthermore, we measured H_2_O_2_ production using the ROS-Glo^™^ H_2_O_2_ Assay with strains AI_2211 and AI_2218 in a 1 ml defined medium. The assay was conducted at shaking speeds of 250 (lower shaking speed) and 500 (higher shaking speed) rpm by RTS-1C to save ROS-Glo^™^ detection reagents (a mini bioreactor with better-shaking speed control) instead of in flasks. Our results indicated H_2_O_2_ concentration to be about 40,000 relative light unit (RLU) for strain AI_2211 shaken at 500 rpm (higher shaking speed), which was 1.6-fold higher than that of strain AI_2211 shaken at 250 rpm (lower shaking speed) and that of strain AI_2218 shaken at both speeds (Fig. 3B). The measurement of H_2_O_2_ concentration demonstrated that at high agitation rates, strain AI_2211 was associated with more oxidative stress by H_2_O_2_. Moreover, it also proved that strain AI_2218 eliminated H_2_O_2_ efficiently because of *ahpC/F* overexpression. Also, the α-ionone production was inversely correlated with the H_2_O_2_ accumulation for strain AI_2211, whereas similar α-ionone titers were obtained for strain AI_2218 when H_2_O_2_ concentration remained low (Fig. 3B). This suggests that indeed H_2_O_2_ negatively affected α-ionone biosynthesis.

### 6-Methyl-5-hepten-2-one (MHO) production in strain AI_2211 and strain AI_2218 under different shaking speed conditions

**Fig. 4:**
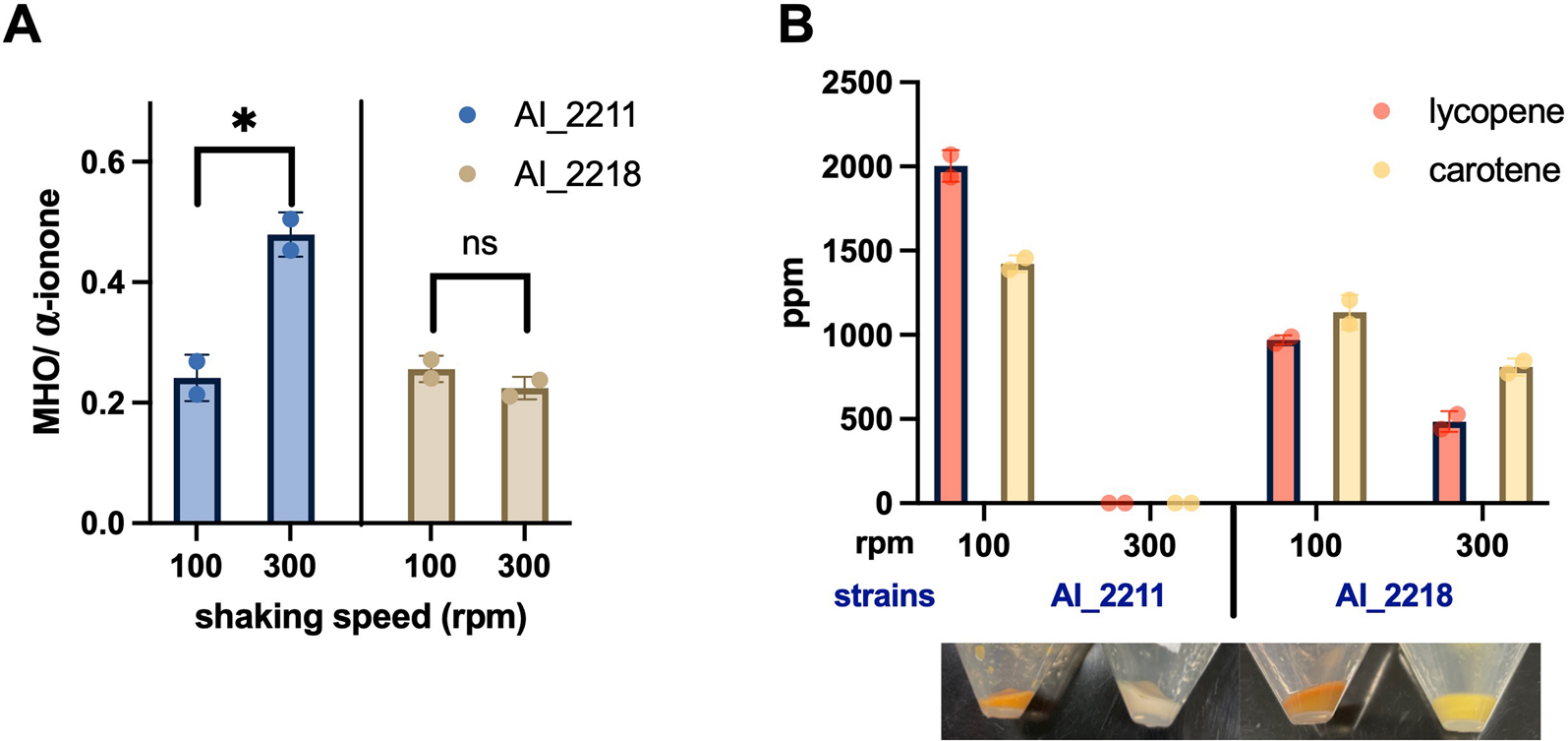
Investigation of the pathway intermediates and side products. **A** The MHO and α-ionone production ratio of strains AI_2211 and AI_2218 under 100 rpm and 300 rpm shaking speed by flask. **B** The composition of carotenoids (lycopene and carotene) for the different-colors cell pellets. The values were statistically analyzed by student’s t-test: ns, P > 0.05; * P < 0.05; ** P < 0.01; *** P < 0.001

Next, we would like to examine how H_2_O_2_ affected the α-ionone pathway and further decreased the production of α-ionone in strain AI_2211. From previous experiments, we observed that the pellet colors of strain AI_2211 were orange and white at shaking speeds of 100 rpm and 300 rpm, respectively. Naturally-occurring lycopene is red and ε-carotene is yellow [8]. Thus, we postulate that the H_2_O_2_ might degrade some intermediates of the pathway, such as lycopene and ε-carotene; this would reduce the pathway flux for α-ionone production. From the experiment with strains AI_2211 and AI_2218 inoculated at shaking speeds of 100 rpm and 300 rpm, we noted the presence of 6-Methyl-5-hepten-2-one (MHO), which is an oxidative degradation product of lycopene [9]. We compared the production of MHO with α-ionone in different cells. The MHO to the α-ionone ratio of strain AI_2211 at 300 rpm was about 0.5 which was two times higher than in all other tests done (around 0.2–0.3) (Fig. 4A). These results indicated that the high shaking speeds led to the high H_2_O_2_ accumulation which degraded the lycopene to MHO in strain AI_2211, and the reduced supply of lycopene further contributed to the decrease in α-ionone production (Fig. 1). Moreover, carotenoid degradation can also be proven from the colorless pellet of strain AI_2211 shaken at 300 rpm (compared to the orange pellet of strain AI_2211 shaken at 100 rpm and the yellow pellet of strain AI_2218 shaken at 300 rpm) (Fig. 4B). For strain AI_2211 at 300 rpm, we couldn’t detect any peak of lycopene and ε-carotene, consistent with its colorless pellets. At 100 rpm,  ~ 2000 ppm lycopene and 1400 ppm ε-carotene were produced in strain AI_2211. For strain AI_2218 at both 100 and 300 rpm, we detected the intracellular ε-carotene and lycopene, although the contents were a bit less than that in strain AI_2211 at 100 rpm (Fig. 4B).

### Strain AI_2218, bioreactor fermentation for α-ionone production

**Fig. 5:**
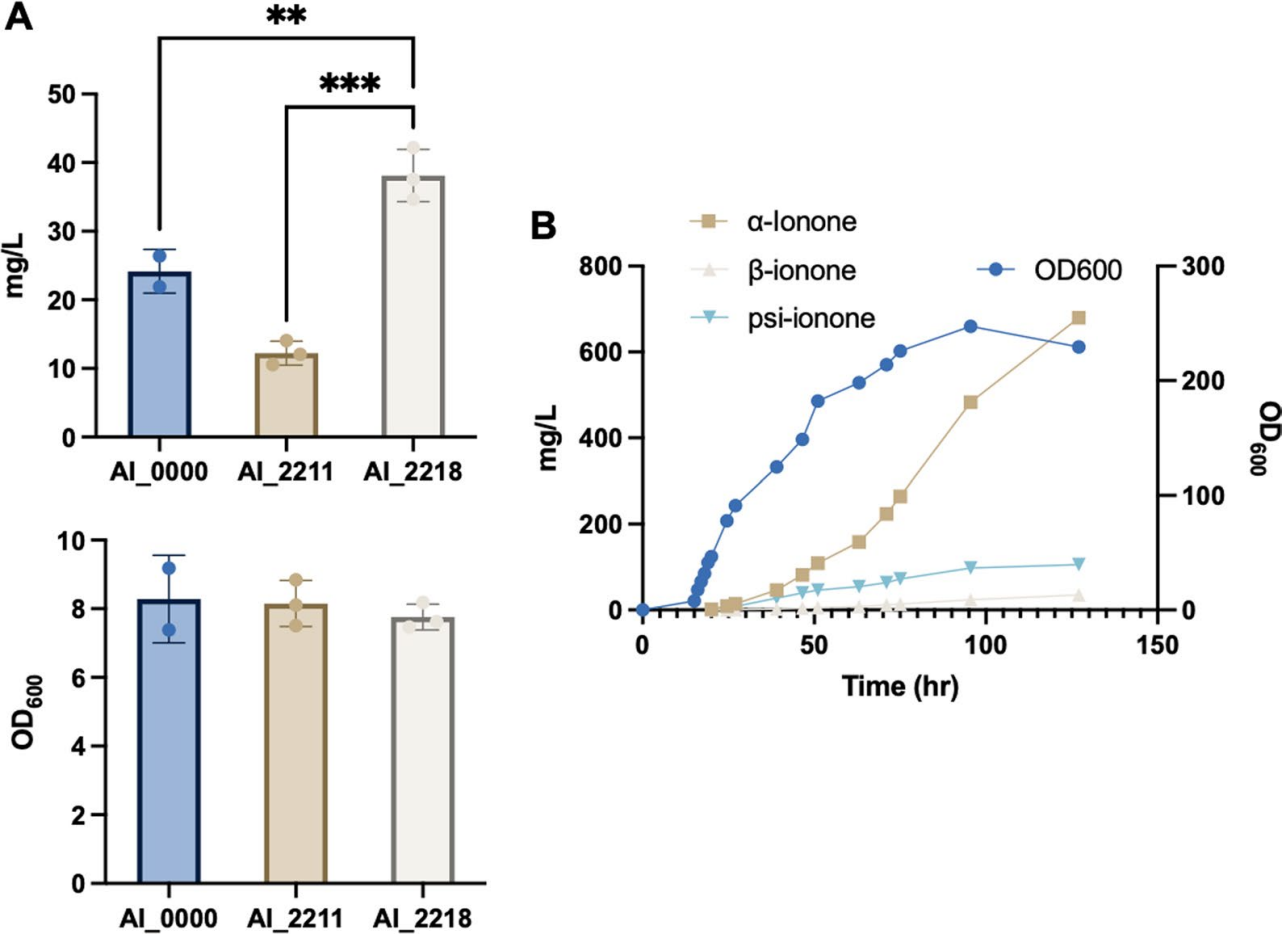
Fed-batch fermentation for α-ionone production. **A** Compare α-ionone titer and OD_600_ by different strains: AI_0000, AI_2211, and AI_2218. All done in 10 ml defined medium with 10 ml dodecane by flask under 300 rpm shaking speed. The titers were statistically analyzed by one-way ANOVA: ns, P > 0.05; * P < 0.05; ** P < 0.01; *** P < 0.001. **B** The fed-batch fermentation of strain AI_2218. Time course profiles of ionone production and cell growth (OD_600_). At the endpoint of 127 h, final OD_600_ was 229.3, and the titers of α-, β- and psi- ionone were 679.9, 35.2 and 105.8 mg/L, respectively

Before scaling up in a 5 L bioreactor, we compared strains AI_0000, AI_2211, and AI_2218 in flasks at 300 rpm to confirm that strain AI_2218 can produce higher α-ionone titers (Fig. 5A). Indeed, strain AI_2218 gave the highest α-ionone yield (5.0 mg/L/OD) as compared to strain AI_0000 (3.0 mg/L/OD) and strain AI_2211 (1.5 mg/L/OD) (Additional file 2). Although strain AI_2211 produced the highest amount of α-ionone under 1 ml defined medium at 300 rpm shaking speed (11.4 mg/L/OD) (Additional file 2), due to its extremely high sensitivity towards oxygen, scaling up using strain AI_2211 is very challenging. In comparison, strain AI_2218 had higher α-ionone yield and higher robustness as less sensitive to oxygen, which is more promising for scaling up. The 5 L bioreactor experiment was thus subsequently performed with strain AI_2218, yielding approximately 700 mg/L α-ionone in 127 h (Fig. 5B), which is about 75% higher than a recent achievement in *Yarrowia lipolytica* [10].

## Discussion

In this study, we improved α-ionone titer with strain AI_2211 by reengineering the biosynthetic pathway and further overexpressing the CCD1 encoding gene. CCD enzymes have been found to be a limiting step in both *E. coli* and yeasts to produce apocarotenoids [10, 11], and their low activities often limit the bioproduction of apocarotenoids [4]. However, our study here indicates that expressing too much CCD1 has a disadvantage that the strain suffers from low stability and high sensitivity to oxygen. This low robustness severely impacted its scaling up; while scaling up from 1 mL test tubes to 10 mL shake flasks, the ionone production dropped significantly from 96 to 19.3 mg/L. Furthermore, we found that lowering the shaking speed from 300 to 100 rpm could partially alleviate the problem. Since shaking flasks have relatively higher oxygen supply than test tubes due to wider space and higher shaking speed leads to higher oxygen supply, we hypothesized that oxidative stress induced by high oxygen supply could be the reason for the drastically decreased yield of strain AI_2211 in flasks (Fig. 2B). Moreover, we showed that the oxidative stress displayed in strain AI_2211 was due to the additional copy of CCD1. Oxidative stress in cells are often the result of ROSs accumulation, which can damage intracellular components (such as lipids, proteins, and DNA) [12]. This led us to speculate that the elevated CCD1 activity could be positively correlated with the accumulation of ROSs [13]. H_2_O_2_ is a major ROS formed during the aerobic respiration process. Our results further indicated that strain AI_2211 produced higher concentrations of H_2_O_2_ at higher shaking speeds (Fig. 3B), which validated our hypothesis that overexpressing too much CCD1 can lead to higher oxidative stress to the cells and reducing the strain robustness. This could be due to the reduction of O_2_ to H_2_O_2_ during CCD1 catalysis, which warrants further studies.

Next, we further confirmed higher amounts of H_2_O_2_ led to faster lycopene degradation and produced a higher amount of side products such as MHO (Fig. 4). This finding is rational as lycopene is a potent antioxidant that has many double bonds and is easily oxidized by ROSs including H_2_O_2_. As the direct precursor to ε-carotene and α-ionone, lycopene degradation further reduced the carbon flux towards α-ionone (Fig. 1). To prevent this, we harnessed *E. coli*’s native mechanisms to mitigate oxidative damage by overexpressing alkyl hydroperoxide reductase (AhpC/F) and the catalase KatG to scavenge excess H_2_O_2_ [14]. When the intracellular H_2_O_2_ concentration is low in *E. coli*, AhpC/F is critical in eliminating H_2_O_2_ [15], but when the intracellular H_2_O_2_ level rises to more than 20 μM, the dominant role shifts to KatG [15]. Our data indicated that AhpC/F was more effective than KatG in reducing H_2_O_2_ and its overexpression in strain AI_2218 resulted in higher α-ionone production at 300 rpm in flask (Fig. 3A) which could be because intracellular H_2_O_2_ concentration was below 20 µM in our ionone strains.

As compared to strain AI_2211, strain AI_2218 has shown improved robustness to oxygen fluctuation and stable α-ionone production in both low and high shaking speeds in flasks. This robustness was successfully translated from 10 mL flasks to 5L bioreactors, in which strain AI_2218 produced approximately 700 mg/L, the highest titer for α-ionone obtained in microbial cell factories reported to date, paving the way for its commercialization. More importantly, we believe the strategy of mediating oxidative stress is inspiring to the biosynthesis of other commercially important apocarotenoids, such as β-ionone, retinol and crocin, in which oxygenases are also involved similarly to CCD1 here.

We also highlighted that the ionone production of AI_2218 in flasks (5.0 mg/L/OD) was still much lower than that of AI_2211 in tubes (11.4 mg/L/OD) (Additional file 2), indicating there are still factors and conditions which we have not explored; identifying these factors and conditions may lead to further increase in α-ionone bioproduction. For instance, in the future, we may further engineer the strain to better balance the CCD1 and AhpC/F activities, so that CCD1 activities are sufficient and H_2_O_2_ production is minimized concurrently.

Lastly, our study reiterates the difficulty in scaling up for some high-yield strains from small scale, due to the differences among tubes, flasks and bioreactors. For example, strain AI_2211 has a very high α-ionone yield in tubes but cannot be scaled up even to 10 mL flasks. As such, we may face a trade-off between yields and robustness. To guarantee stable outputs and product quality in industrial production, we may have to choose a strain with higher robustness but a relatively lower yield such as strain AI_2218.

## Conclusion

CCD1 is known to be the limiting enzyme for α-ionone biosynthesis. Here, we increased CCD1 activities by introducing an extra copy of CCD1 in a plasmid, which did improve ionone production in shake tubes. However, the increased CCD1 activities also reduced strain stability and elevated the oxidative stress as shown in the accumulation of higher amount of H_2_O_2_. H_2_O_2_ accelerated the degradation of lycopene (the precursor to α-ionone) and reduced the flux towards α-ionone while scaling up in flasks. Therefore, we overexpressed *ahpC/F* which successfully removed H_2_O_2_ and prevented the rapid degradation of lycopene, leading to a further increase in α-ionone titer and higher robustness in flasks. To the best of our knowledge, this is the first study that investigated oxidative stress induced by CCD1 and its effect on α-ionone production, which is inspiring to the biosynthesis of other commercially important apocarotenoids, such as β-ionone, retinol and crocin. Finally, our best strain produced 700 mg/L (here, the titre was calculated based on the final volume of aqueous phase) α-ionone in a 5 L bioreactor, which was the highest in the literature, paving the way for its commercialization.

## Methods

### Bacteria strains, plasmids, and oligonucleotides

The bacterial strains and plasmids used in this study are listed in Additional file 1. The specific oligonucleotides used for PCR amplification were synthesized by Integrated DNA Technologies (IDT) and listed in Additional file 3. We constructed Modules 4–7 (p15A-amp-$\Delta$N50LsLCYe-OfCCD1-trxA (TM1) with three mutations of OfCCD1 active site loop, adding *katG*) and 4–8 (p15A-amp-$\Delta$N50LsLCYe-OfCCD1-trxA (TM1) with three mutations of OfCCD1 active site loop, adding *ahpC/F*). The backbone was amplified with Module 4–1 as a template and the genes of the *katG* and *ahpC/F* were amplified from *E. coli* BL21. Cloning was performed using the iProof ™ High-Fidelity DNA Polymerase (BIO-RAD).

### Media and culture conditions

All the cells were grown in LB media [16]. For the production test, a chemically defined auto-induction medium was used, which contained 2 g/L glucose and 8 g/L glycerol, 2 g/L ammonium sulfate, 4.2 g/L KH_2_PO_4_, 11.24 g/L K_2_HPO_4_, 1.7 g/L citric acid, 0.5 g/L MgSO_4_, and 10 mL/L trace element solution. The trace element solution (100 x) contained 0.25 g/L CoCl_2_·6H_2_O, 1.5 g/L MnSO_4_·4H_2_O, 0.15 g/L CuSO_4_·2H_2_O, 0.3 g/L H_3_BO_3_, 0.25 g/L Na_2_MoO_4_·2H_2_O, 0.8 g/ L Zn(CH_3_COO)_2_, 5 g/L Fe(III) citrate, and 0.84 g/L ethylenediaminetetraacetic acid (EDTA) at pH 8.0. The auto-induction medium was supplemented with the appropriate antibiotics (100 mg/L ampicillin, 34 mg/L chloramphenicol, 50 mg/L kanamycin, and 50 mg/L spectinomycin) to maintain corresponding plasmids. Cells were induced by 15 mM lactose [17]. 1% fresh cell culture was inoculated into 1 ml of auto-induction medium in 14 ml snap cap tubes and 10 ml of auto-induction medium in 100 ml flasks. After induction, dodecane (200 μL for the 1 ml culture and 10 ml for the 10 ml culture) was added to the culture to extract ionone, and the cells were incubated at 28 °C for 72 h with a shaking speed of 100 rpm or 300 rpm before harvest.

### Quantification of α-ionone and 6-Methyl-5-hepten-2-one (MHO)

The α-ionone, or MHO samples were prepared by diluting 10–50 times of organic layer into 1000 μL hexane. Gas chromatography–mass spectrometry (GC–MS) analyses of the samples were performed on an Intuvo 9000 GC system attached with a 5977B MS detector (Agilent Technologies, USA). The system was equipped with a polar DB wax column (polyethylene glycol (PEG); 30 m × 0.25 mm I.D. × 0.25 µm: Agilent Technologies, USA) and a split injector (split ratio 1:10). The oven program started at 80 °C for 1 min, with temperature increased by 20 °C/min until 130 °C, held for 1.5 min, before being increased by 40 °C/min until 200 °C. This was held for 2 min, before being finally increased by 80 °C/min and maintained at 230 °C for another 2 min. Helium was used as the carrier gas at a constant flow rate of 1.0 mL/min. The Agilent 5977B mass spectrometer was operated in the electron ionization mode at 70 eV with a source temperature of 230 °C, transfer line temperature set at 250 °C, and a scan range of m/z 50–500 in the full scan mode at an acquisition rate of 3.6 scans/s. Methanol was the solvent for the column wash and hexane for the needle wash. The injection volume was 1 µL. The ionone concentrations were calculated by interpolating with a standard curve prepared by commercial standards (Sigma-Aldrich Pte Ltd, Singapore). The mass spectrometer was operated in EI mode with a full scan analysis.

### ROS-Glo^™^ H_2_O_2_ Assay

ROS-Glo (Promega, Madison, WI, USA) assay was used as specified to quantify H_2_O_2_ production [18, 19]. *E. coli* strains AI_2211 and AI_2218 were inoculated into 1 ml auto-induced R-medium by RTS-1C (Personal Bioreactor, Biosan). Cells were grown to an OD_600_ of 1.5–2.0 and then the H_2_O_2_ substrate was added. The culture was subsequently incubated for 1.5 h, the cells harvested, and the amount of luciferin precursor produced measured. The H_2_O_2_ substrate reacts directly with H_2_O_2_ to generate a luciferin precursor. Upon addition of ROS-Glo^™^ Detection Reagent containing Ultra-Glo^™^ Recombinant Luciferase and d-Cysteine, the precursor is converted to luciferin by the d-Cysteine, and the luciferin produced reacts with Ultra-Glo^™^ Recombinant Luciferase to generate a luminescent signal that is proportional to H_2_O_2_ concentration (ROS-Glo^™^ H_2_O_2_ Assay, Promega). 800 µL of cells were incubated with 200 µL of H_2_O_2_ substrate followed by the addition of the ROS-Glo detection reagent. Luminescence corresponding to H_2_O_2_ levels was measured using a micro plate reader (Molecular Devices, San Jose, CA, USA).

### Quantification of carotenoids

Intracellular carotenoids were extracted from cellular pellets according to the previous method [4]. Briefly, 10–50 μL bacterial culture was collected and centrifuged. Cell pellets were washed with PBS and were resuspended in 20 μl of water, followed by addition of 180 μl of acetone. The high performance liquid chromatography (HPLC) method was modified as previously described. Briefly, the analysis employed an Agilent 1260 Infinity LC System equipped with a ZORBAX, Eclipse Plus C18, 4.6 × 250 mm, 5 μm column and diode array detector (DAD). Isocratic condition (50% methanol, 48% ethyl acetate, and 2% water) was maintained at 1.5 mL/min for 5 min. The carotenoids were detected at wavelength of 450 nm. Standard curves were generated using commercial standards of ε-carotene (CaroteNature, Switzerland) and lycopene (Santa Cruz Biotechnology, Dallas, TX).

### Fed-batch fermentation

The starting medium was a chemically defined medium modified from previous studies [17], which contained 5 g/L of glucose transferred into a 5L bioreactor with an initial working volume of 1.8L. The *E. coli* strain AI_2218 was inoculated into the sterile defined medium to obtain an initial optical density at 600 nm (or OD_600_) of 0.1. The fermentation was first carried out under the controlled set points of pH, temperature, and dissolved oxygen at 7.0, 37 °C, and 30%, respectively. After inoculation, peristatic pumping of the feedstock solution (containing 750 g/L glucose and 7.5 g/L MgSO_4_) at 1.62 mL/h flow rate was carried out overnight (~ 13 h). The pH of the culture was controlled at 7.0 using an alkaline solution (a mixture of 28% ammonium hydroxide and 1 M sodium hydroxide solution; in ratio 1:1 by volume) throughout the experiments. The flow rate of feedstock was changed to an exponential feeding rate after 13 h, cells were induced by 0.1 mM Isopropyl β-d-1-thiogalactopyranoside (IPTG) when OD_600_ reached about 40.0 and 700 mL of sunflower oil as an extractant was then added to the bioreactor. Dissolved oxygen was subsequently lowered to 15% and the flow rate of the feedstock was kept constant at 15.6 mL/h after induction.

## Supplementary Information

**Additional file 1.** Strains and plasmids used in the study.

**Additional file 2.** Content (Titer/OD600) of different α-ionone producing strains.

**Additional file 3.** Oligonucleotides used for PCR amplifications.

## Data Availability

All the data analyzed in this study are included in this manuscript.

## Abbreviations

CCD1: Carotenoid cleavage dioxygenase 1
H_2_O_2_: Hydrogen peroxide
ROSs: Reactive oxygen species
*ahpC/F*: Alkyl hydroperoxide reductase
MHO: 6-Methyl-5-hepten-2-one
