# Supplementary material for: Mediating oxidative stress enhances α-ionone biosynthesis and strain robustness during process scaling up
Source: Microb Cell Fact. 2022 Nov 23;21:246. doi: 10.1186/s12934-022-01968-1 (PMC9686065; doi:10.1186/s12934-022-01968-1)
Supplement: Supplementary file 1 — Additional file 1. Strains and plasmids used in the study. [file 12934_2022_1968_MOESM1_ESM.docx]

**Mediating oxidative stress enhances α-ionone biosynthesis and strain robustness during process scaling up**

Ching-Ning Huang^1^, Xiaohui Lim^1^, Leonard Ong^1^, Chin Chin Lim^1^, Xixian Chen^1*^ and Congqiang Zhang ^1*^.

^1^Singapore Institute of Food and Biotechnology Innovation (SIFBI), Agency for Science, Technology and Research (A*STAR), 31 Biopolis Way Level 6 Nanos building Singapore 138669.

* Corresponding author:

Xixian Chen and Congqiang Zhang

E-mail: [xixian_chen@sifbi.a-star.edu.sg](mailto:xixian_chen@sifbi.a-star.edu.sg) and [zcqsimon@outlook.com](mailto:congqiang_zhang@sifbi.a-star.edu.sg)

^1^Singapore Institute of Food and Biotechnology Innovation (SIFBI), Agency for Science, Technology and Research (A*STAR).

**Additional files**

**Additional file 1: Strains and plasmids used in the study**

| Strains | Describe of plasmids | | Sources |
| --- | --- | --- | --- |
| ***E. coli* BL21 DE3** $\boldsymbol{\Delta}$***aro*ABC**$\boldsymbol{\Delta}$***ser*C** | *E. coli* BL21-Gold DE3 strain  deletes the gene *aro*A, *aro*B, *aro*C and *ser*C | | This study |
| **AI_0000** | Module 1: p15A-spec-hmgS-atoB-hmgR (TM1)  Module 2: p15A-cam-mevK-pmk-pmd-idi (TM2)  Module 3: p15A-kan-crtEBI-ispA (TM1)  Module 4: p15A-amp-$\Delta$N50LsLCYe-OfCCD1-trxA (TM1) | | [1] |
| **AI_2211** | Module 1: p15A-spec-hmgS-atoB-hmgR- OfCCD1-trxA (TM2)  Module 2: p15A-cam-mevK-pmk-pmd-idi (TM3)  Module 3: p15A-kan-crtBI-ispA-MbGGPPs (TM1)  Module 4: p15A-amp-$\Delta$N50LsLCYe-OfCCD1-trxA (TM1) with three mutations of OfCCD1 active site loop [2] | | This study |
| **AI_3211** | | Module 1: p15A-spec-hmgS-atoB-hmgR (TM2)  Module 2: p15A-cam-mevK-pmk-pmd-idi (TM3)  Module 3: p15A-kan-crtBI-ispA-MbGGPPs (TM1)  Module 4: p15A-amp-$\Delta$N50LsLCYe-OfCCD1-trxA (TM1) with three mutations of OfCCD1 active site loop (6) | This study |
| **AI_2217** | | Module 1: p15A-spec-hmgS-atoB-hmgR- OfCCD1-trxA (TM2)  Module 2: p15A-cam-mevK-pmk-pmd-idi (TM3)  Module 3: p15A-kan-crtBI-ispA-MbGGPPs (TM1)  Module 4: p15A-amp-$\Delta$N50LsLCYe-OfCCD1-trxA (TM1) with three mutations of OfCCD1 active site loop, adding *katG*. | This study |
| **AI_2218** | | Module 1: p15A-spec-hmgS-atoB-hmgR- OfCCD1-trxA (TM2)  Module 2: p15A-cam-mevK-pmk-pmd-idi (TM3)  Module 3: p15A-kan-crtBI-ispA-MbGGPPs (TM1)  Module 4: p15A-amp-$\Delta$N50LsLCYe-OfCCD1-trxA (TM1) with three mutations of OfCCD1 active site loop, adding *ahpC/F*. | This study |
