# Supplementary material for: Mediating oxidative stress enhances α-ionone biosynthesis and strain robustness during process scaling up
Source: Microb Cell Fact. 2022 Nov 23;21:246. doi: 10.1186/s12934-022-01968-1 (PMC9686065; doi:10.1186/s12934-022-01968-1)
Supplement: Supplementary file 2 — Additional file 2. Content (Titer/OD600) of different α-ionone producing strains. [file 12934_2022_1968_MOESM2_ESM.docx]

**Mediating oxidative stress enhances α-ionone biosynthesis and strain robustness during process scaling up**

Ching-Ning Huang^1^, Xiaohui Lim^1^, Leonard Ong^1^, Chin Chin Lim^1^, Xixian Chen^1*^ and Congqiang Zhang ^1*^.

^1^Singapore Institute of Food and Biotechnology Innovation (SIFBI), Agency for Science, Technology and Research (A*STAR), 31 Biopolis Way Level 6 Nanos building Singapore 138669.

* Corresponding author:

Xixian Chen and Congqiang Zhang

E-mail: [xixian_chen@sifbi.a-star.edu.sg](mailto:xixian_chen@sifbi.a-star.edu.sg) and [zcqsimon@outlook.com](mailto:congqiang_zhang@sifbi.a-star.edu.sg)

^1^Singapore Institute of Food and Biotechnology Innovation (SIFBI), Agency for Science, Technology and Research (A*STAR).

**Additional file 2: Content (Titer/OD600) of different α-ionone produce strains.**

**The content of Fig. 2B.**

**The content of Fig. 5A.**

**Reference**

1. Zhang C, Chen X, Lindley ND, Too HP. A “plug‐n‐play” modular metabolic system for the production of apocarotenoids. Biotechnol. Bioeng. 2018; 115:174-83.

2. Chen X, Shukal S, Zhang C. Integrating enzyme and metabolic engineering tools for enhanced α-ionone production. J. Agric. Food Chem. 2019; 67:13451-59.
